# A Rare Post-infectious Rash: Pityriasis Rubra Pilaris After COVID-19 Infection

**DOI:** 10.7759/cureus.43810

**Published:** 2023-08-20

**Authors:** Philicia Duncan, Daniel Flood, Christina Dietz

**Affiliations:** 1 Division of Hospital Medicine, The Ohio State University Wexner Medical Center, Columbus, USA; 2 Department of Dermatology, The Ohio State University Wexner Medical Center, Columbus, USA

**Keywords:** hospital based medicine, inflammatory skin disorder, covid 19, erythroderma, pityriasis rubra pilaris

## Abstract

Pityriasis rubra pilaris (PRP) is a rare papulosquamous skin disorder that often presents with erythematous follicular-based hyperkeratotic papules that can become confluent and lead to erythroderma and electrolyte and thermoregulatory imbalances resulting from increased tissue perfusion and skin barrier breakdown. Due to this condition being uncommon, many specialties outside of dermatology are unfamiliar with this entity which poses unique diagnostic and management challenges. This case report involves a 55-year-old woman who presented to the emergency room with erythroderma secondary to PRP. It highlights the relevance of PRP in the context of in-hospital management by presenting the patient’s clinical profile, diagnostic workup, and treatment plan. By emphasizing the distinctive clinical features and natural course of the disease, this report aims to enhance the understanding of this uncommon inflammatory skin condition.

## Introduction

Pityriasis rubra pilaris (PRP) is a rare inflammatory dermatosis. Although PRP was first described in 1835, the etiology and pathogenesis remain largely unknown [1]. However, this condition has been associated with infection, vaccination, malignancy, UV exposure, autoimmune disorders, and trauma [1-4]. Given PRP has debilitating sequelae that frequently result in poor quality of life and depression, early recognition and diagnosis are crucial [5]. PRP has been classified into six subtypes, based on Griffith’s classification, with variable presentations and natural history. Though PRP may be confused with other erythrodermic disorders, there are characteristic physical and histologic findings that aid in diagnosis. We described a rare case of a 55-year-old female presenting with new-onset PRP three weeks after COVID-19 infection. This article was previously presented as a poster at the 2023 Society of Hospital Medicine Annual Meeting on March 28, 2023.

## Case presentation

The patient was a 55-year-old woman with latent autoimmune diabetes in adults well managed with insulin (HbA1C: 6.2%), diagnosed four years prior to presentation. There was no prior history of rheumatologic disease or skin disorders and the patient presented to the hospital with erythroderma, chills, diffuse pruritus, and severe pain in her hands and feet. Two months prior to presentation, she had a sudden eruption of patchy, erythematous plaques and diffuse scaling of the scalp, face, upper chest, and back. Notably, approximately three weeks prior to the onset of the rash, she was diagnosed with mildly symptomatic COVID-19 infection.

She was seen in the outpatient dermatology clinic. The laboratory examination revealed a slight leukocytosis of 12.12 K/uL without a left shift, normal liver, and renal function, positive antinuclear antibody (ANA) of 1:320, negative double stranded DNA (DsDNA), extractable nuclear antigen (ENA), and hepatitis viral panel. A skin biopsy was performed. The biopsy demonstrated interface dermatitis with negative direct immunofluorescence. These non-specific findings led to an incorrect diagnosis of subacute cutaneous lupus erythematosus and started on oral prednisone, hydroxychloroquine, and mycophenolate Her rash failed to improve after six weeks of therapy, prompting a repeat biopsy. This second biopsy revealed psoriasiform dermatitis with alternating ortho- and parakeratosis with follicular plugging, findings consistent with a diagnosis of pityriasis rubra pilaris. Her rash progressed to involve her entire torso and extremities. As a result of the progression, outpatient dermatology advised hospital admission to evaluate for electrolyte abnormalities, thermoregulation problems, or other potential complications.

**Figure 1:**
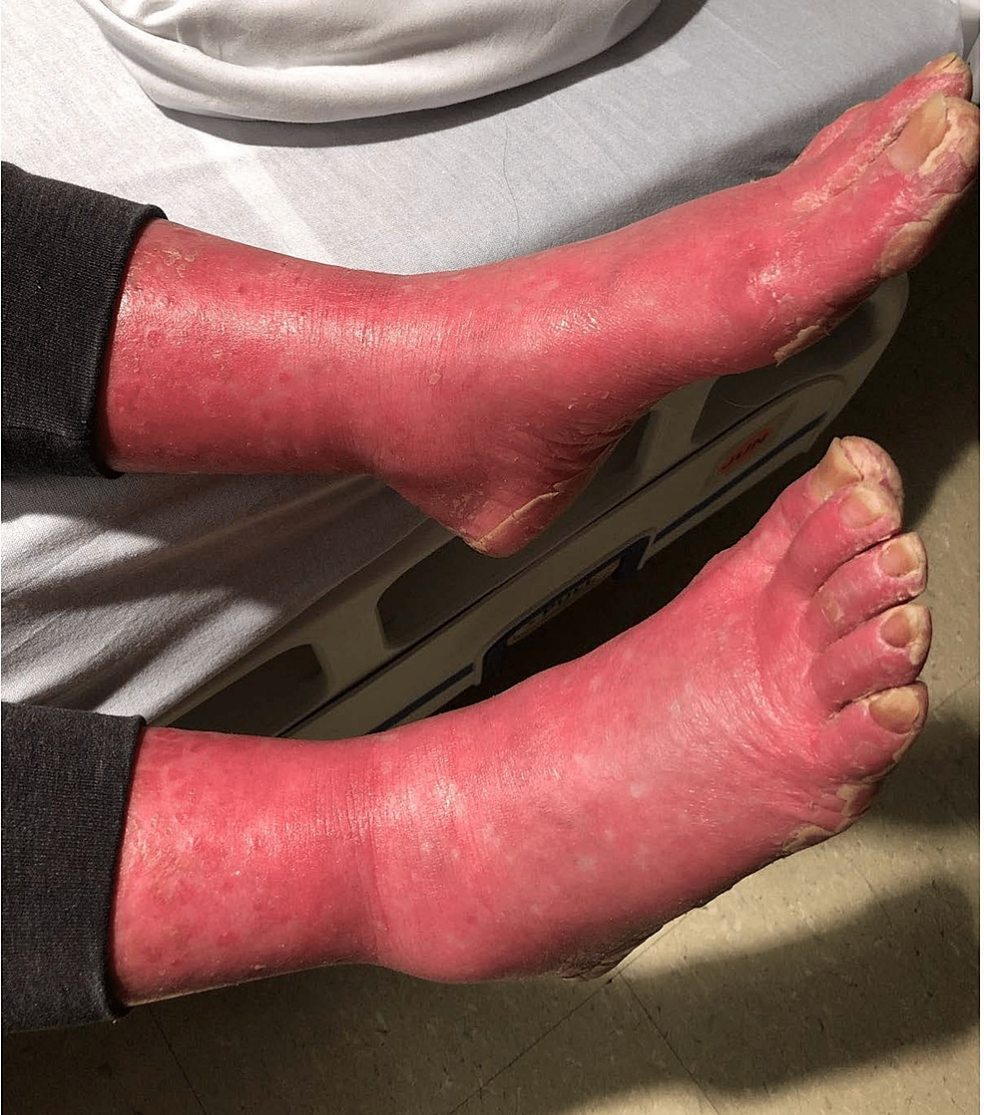
Erythroderma

**Figure 2:**
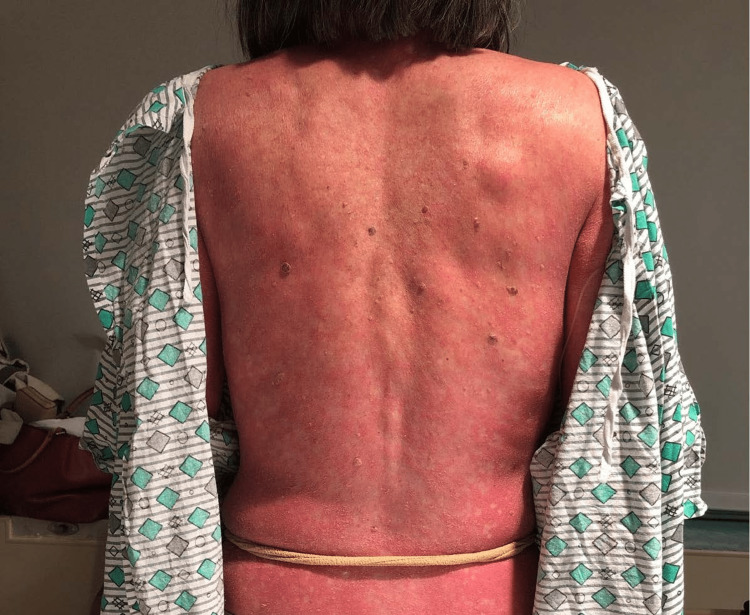
Erythroderma with “islands of sparing”

**Figure 3:**
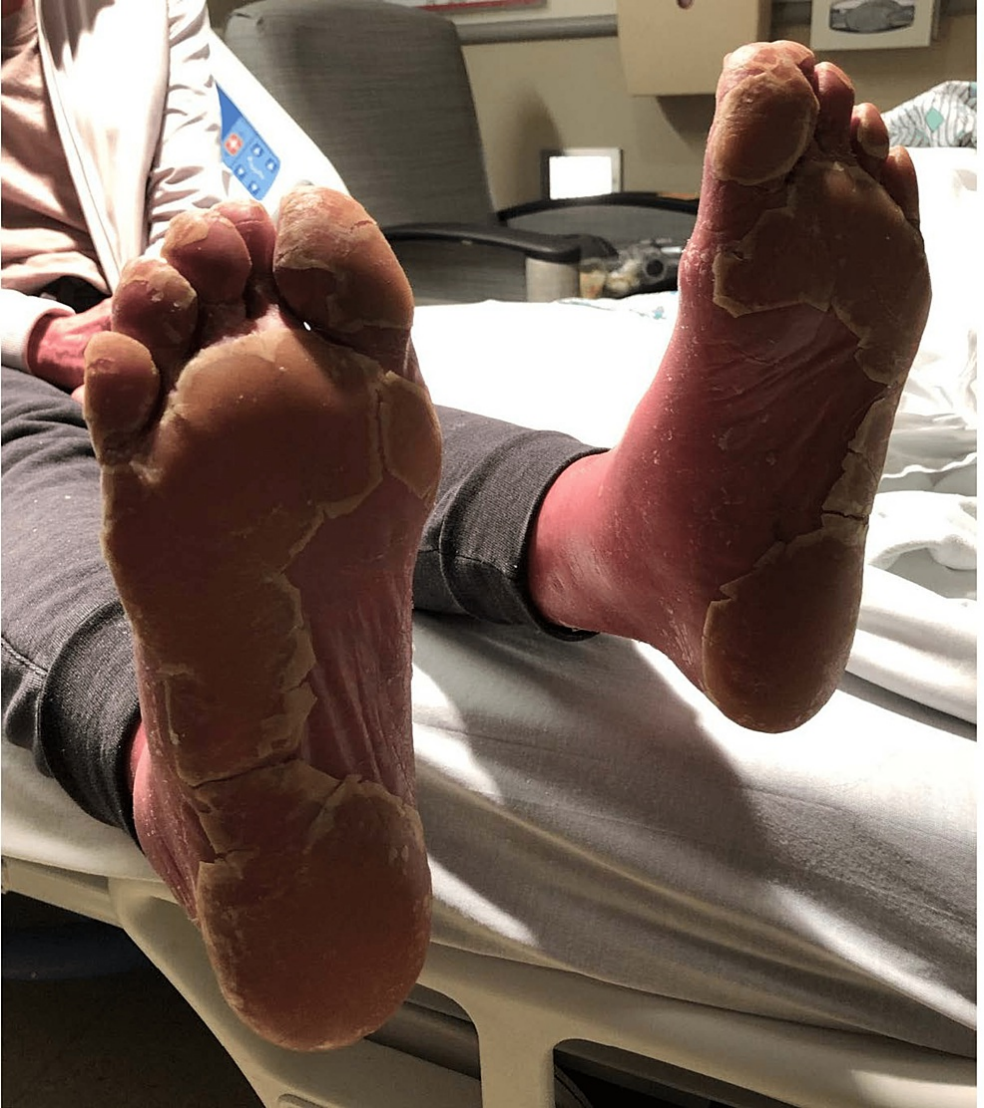
Prominent keratoderma with yellowish discoloration

On physical exam at the hospital, the patient had normal vital signs and unremarkable cardiac, lung, and abdominal examinations. Her physical exam was notable for diffuse erythema encompassing approximately 90% of her body surface area, erythematous plaques with islands of sparing (Figures 1, 2), and waxy keratoderma of the palms and soles (Figure 3). Dermatology was consulted to aid in diagnosis and management. Erythematous plaques with islands of sparing as well as palmar and plantar waxy keratoderma are specific findings associated with PRP. These skin changes coupled with the pathognomonic findings of alternating ortho- and parakeratosis with follicular plugging on skin biopsy led to a diagnosis of pityriasis rubra pilaris. Consequently, she was started on acitretin, topical triamcinolone, and a prednisone taper. There are no randomized control trials regarding systemic therapy in PRP, though oral retinoids such as Acitretin have shown efficacy in this condition. She was discharged from the hospital on this regimen with outpatient dermatology follow-up.

One month after discharge from the hospital, the patient was seen in the outpatient dermatology clinic without significant improvement in symptoms. Therefore, ixekizumab was added to the acitretin with monthly dermatology visits and laboratory evaluation with complete blood count (CBC) and comprehensive metabolic panel (CMP). After three months of ixekizumab and acitretin, she developed alopecia with modest improvement in her skin findings and stable laboratory findings. Her alopecia was believed to be partially related to acitretin, so this medication was discontinued. Unfortunately, she then developed a methicillin-resistant Staphylococcus aureus (MRSA) skin and soft tissue infection and recurrent injection site reactions. These complications made it difficult for the patient to tolerate continued injections. As a result, she was treated with the appropriate antibiotics for the MRSA infection and the ixekuzimab injections were discontinued. She was subsequently started on cyclosporine and is improving, but still has disease activity. She has ongoing monitoring and treatment adjustment with dermatology.

## Discussion

This is a unique case because while there are many case reports of PRP presenting after COVID-19 vaccination, there are few cases of PRP after a recent COVID-19 infection [6,7]. As stated in the introduction, little is certain about the etiology of PRP, and the pathogenesis remains unknown [1,8]. PRP has been found to be associated with infection, vaccine, malignancy, UV exposure, medications, and trauma [3].

PRP is commonly acquired, but also can rarely be an inherited condition. It has a bimodal distribution where the condition is most commonly seen in the first or the sixth decade of life [1.3]. Classic adult PRP, as seen in our patient, begins on the head and neck and progresses caudally. Follicular hyperkeratosis on an erythematous base is a key feature [1,3]. This finding has been described as resembling a nutmeg grater [3]. When these hyperkeratotic papules coalesce into plaques on the trunk and extremities, they often leave distinctive “islands of sparing” [3]. The palms and soles commonly have orange-red waxy keratoderma [3]. Many patients will progress rapidly after the onset of the disease to erythroderma [3]. Nail involvement may also be seen and is characterized by yellow-brown discoloration and subungual debris [3]. Mucous membranes are rarely involved [3].

The Griffiths classification is the commonly used schema to categorize different subtypes of PRP. Types I, II, and VI are seen in adults, while types III, IV, V, and VI are seen in children. All types, except type IV, present with a generalized eruption. Type I, also called the classic adult form, is the most common form of PRP, accounting for around 55% of cases [1]. Type II, or the atypical adult form, accounts for about 5% of cases [1]. The last adult type of PRP, type VI or HIV-associated, accounts for <1% of cases [3]. It is of note that type I PRP, as described in the above case, is often refractory to treatment but is generally self-limited and clears in 3 years in about 80% of patients [1]. It presents as described above.

To diagnose a patient with PRP, a skin biopsy is performed. Histopathology will demonstrate psoriasiform dermatitis with irregular hyperkeratosis and alternating vertical and horizontal ortho- and parakeratosis [1,9]. This has been described as having a “checkerboard” appearance. Follicular plugging and parakeratosis at the edge of hair follicles are also frequently seen [1,3].

There are several conditions that can present with erythroderma causing the determination of the symptom to be challenging. When available, workup should include assistance from dermatology. The main differential diagnosis for PRP is psoriasis. The distinct orange-red waxy palmoplantar keratoderma, follicular prominence with a nutmeg grater appearance, and classic islands of sparing can help guide you to a diagnosis. Also, the absence of an oil drop sign, pitting, and onycholysis of the nails can help differentiate the two entities [1,3]. The differential diagnosis also includes subacute lupus erythematous (SCLE). PRP generally presents with a papulosquamous eruption on the head and neck area, similar to SCLE. In our patient, her original biopsy demonstrated interface dermatitis, consistent with SCLE. As her disease progressed, she developed the classic findings of PRP listed above which helped guide us to the proper diagnosis. Additionally, repeat biopsies were consistent with PRP, not SCLE.

Treatment for PRP remains challenging. Without a unifying etiology and little being known about the pathogenesis, targeted therapy is not possible. First-line therapy is generally a retinoid, such as isotretinoin or acitretin. Methotrexate and other immunosuppressants have also been used either alone or in conjunction with a retinoid. Newer medications such as TNF inhibitors, IL-17 inhibitors, and IL-23 inhibitors have been shown to provide variable benefits [1,3,8,9].

Since PRP is a rare disease, the etiology, prevalence, and pathogenesis of this condition remain unknown. This case highlights an association between a COVID-19 infection and PRP. More research is needed looking into inciting factors of this complicated disease and its association with COVID-19 along with other viral infections.

## Conclusions

While hospitalists might be familiar with various post-infectious, drug-induced, and auto-immune cutaneous manifestations, PRP is a rare skin condition that is not commonly considered in patients presenting with rash after COVID-19 infection. Given that it is frequently misdiagnosed as systemic cutaneous lupus or psoriasis, knowledge of its characteristic physical examination and histopathologic features is important.
